# First Report and Comparative Genomic Analysis of a *Mycoplasma mycoides* Subspecies *capri* HN-A in Hainan Island

**DOI:** 10.3390/microorganisms10101908

**Published:** 2022-09-26

**Authors:** Zhenxing Zhang, Junming Jiang, Meirong He, Haoyang Li, Yiwen Cheng, Qi An, Si Chen, Li Du, Churiga Man, Qiaoling Chen, Lianbin Li, Fengyang Wang

**Affiliations:** Hainan Key Laboratory of Tropical Animal Reproduction & Breeding and Epidemic Disease Research, Engineering Key Laboratory of Haikou, College of Animal Science and Technology, Hainan University, Haikou 570228, China

**Keywords:** *Mycoplasma mycoides* subspecies *capri*, virulence factor, Hainan Island, comparative genomics, gene islands

## Abstract

*Mycoplasma mycoides* subspecies *capri* (Mmc) is one of the six *Mycoplasma mycoides* cluster (Mm cluster) members, which can cause “MAKePS” (Mastitis, Arthritis, Keratoconjunctivitis, Pneumonia, Septicemia) syndrome in ruminants. These symptoms can occur alone or together in individuals or flocks of goats. However, little is known about the epidemic Mmc strains in Hainan Island, China. We aimed to isolate the endemic Mmc strains in Hainan Island and reveal their molecular characteristics by genomic sequencing and comparative genomics to mitigate the impact of Mmc on local ruminant farming. Here, the Mmc HN-A strain was isolated and identified for the first time in Hainan Island, China. The genome of Mmc HN-A was sequenced. It contains a 1,084,691 bp-long circular chromosome and 848 coding genes. The genomic analysis of Mmc HN-A revealed 16 virulence factors, 2 gene islands, and a bacterial type IV secretion system protein VirD4. Comparative genomics showed that the core genome of the five *Mycoplasma mycoides* contained 611 genes that could be exploited to develop drugs and endemic vaccines. Additionally, 36 specific genes were included in the Mmc HN-A genome, which could provide the possibility for the further control and prevention of the Mmc effects on local ruminants and enrich the information on Mmc strains.

## 1. Introduction

Mycoplasmas are the smallest prokaryotic microorganisms without a cell wall and have various filamentous or branched forms. They can infect animals and plants and cause a variety of diseases that negatively affect human health. To date, over 200 mycoplasmas have been discovered, among which over 40 can infect ruminants [1]. Among these mycoplasmas, *Mycoplasma capricolum* subspecies *capripneumoniae* (Mccp), *Mycoplasma mycoides* subspecies *capri* (Mmc), and *Mycoplasma mycoides* subspecies *mycoides* small colony (MmmSC) are important pathogenic bacteria that pose serious health threats and heavy economic losses to the ruminant breeding industry [2].

As we all know, MmmSC can cause respiratory diseases in cattle. It is the causative agent of contagious bovine pleuropneumonia (CBPP), and the initiation of pneumonia leads to the loss of trade and production between regions. As soon as the disease is recognized, the World Organization for Animal Health (OIE) needs to be notified immediately [3,4]. Mccp is highly contagious and can often trigger symptoms such as fibrous pleuropneumonia and pleural fluid accumulation in the thorax of goats. It causes shortness of breath and an uninterrupted cough in goats during the acute onset period [5,6]. Mccp causes contagious goat pleuropneumonia (CCPP), a disease with high morbidity and mortality in goat farming. Mmc can cause similar clinical symptoms and has been mistaken as the CCPP causative agent for a long time [7]. Therefore, clinical isolation and the identification of Mmc help to explore the differences between them.

Mmc can cause “MAKePS” (Mastitis, Arthritis, Keratoconjunctivitis, Pneumonia, Septicemia) syndrome in ruminants [8]. In order to better distinguish and prevent CCPP caused by Mccp and reduce the loss of the goat industry, Thiaucourt et al. defined other respiratory mycoplasma diseases in small ruminants with the same symptoms as “MAKePS” syndrome. It can not only be caused by Mmc, *Mycoplasma mycoides* subspecies *mycoides* large colony (MmmLC), and *Mycoplasma capricolum* subspecies *capricolum* (Mcc) of the *Mycoplasma mycoides* cluster (Mm cluster) but also by *Mycoplasma putrefaciens* (Mp) and *Mycoplasma agalactiae* (Ma) [9]. Additionally, Mmc-infected animals develop symptoms such as increased body temperature, dyspnea, and increased nasal secretions. Mmc has lower morbidity and mortality rates than Mccp-triggered CCPP. However, Mmc outbreaks have been successively reported in recent years [10]. Shah et al. [11] reported a significant increase in Mmc incidence in northern Khyber Pakhtunkhwa. The cold climate decreased the immunity of animals, making them more susceptible. Thousands of goats were infected with Mmc in Mexico, and the Mmc outbreak was associated with approximately 20% morbidity and 10% mortality [12]. In the USA, Kinde et al. [13] studied the Mmc outbreak in California and pointed out that the high mortality rate of Mmc in captive dairy goat kids was due to contaminated colostrum or milk.

Since the whole genome sequence of the Mmc GM12 strain was available in 2009 [14], a total of 25 complete Mmc genomes have been released according to the National Center for Biotechnology Information (NCBI, 24 June 2022), which can open new avenues for comparative and evolutionary genomic studies [15]. However, 23 of them were the whole genome sequences of the Mmc GM12 strain and its mutant strain, and their genomic symmetric identities were all more than 99% (NCBI, 24 June 2022). In addition to the Mmc GM12 strain, only the whole-genome sequences of the MmmLC 95010 and Mmc Ker. TCR. LT strains were available. However, there are still no detailed reports on Mmc genome analysis.

To further explore the pathogenic mechanism of Mmc, the Mmc HN-A strain was isolated from Hainan Island, and whole-genome sequences were obtained. At the same time, we selected the other four *Mycoplasma mycoides* strains from different regions and compared their genomes with the Mmc HN-A strain. These included the Mmc GM12 strain isolated from the United States, the Mmc Ker.TCR. LT strain isolated from India, the MmmLC 95010 strain isolated from France, and the MmmSC PG1 strain isolated from Sweden. This can help us understand the molecular characteristics of the Mmc HN-A strain, such as genetics and virulence factors. It can also promote the clinical diagnosis and the local vaccine development of Mmc. Furthermore, comparative genomic analysis between Mmc and other different subspecies of *Mycoplasma mycoides* helps to screen out important drug targets and provide references for preventing Mmc outbreaks.

## 2. Materials and Methods

### 2.1. Isolation and Identification of Mmc HN-A

A two-month-old female Hainan black goat on a farm in Hainan Province appeared to have symptoms, such as inability to stand, aggravated respiratory sounds, lack of energy, and eating difficulties. The goat was dissected, and samples were collected within 30 min of death. The goat’s elbow was cystic and filled with fluid. A sterile syringe was used to aspirate joint effusion for isolation and identification.

The composition of 100 mL of mycoplasma complete medium was as follows: 2.1 g of PPLO broth (excluding crystal violet) (Difco, Tucker, GA, USA), 0.3 g of glucose (Difco, Tucker, GA, USA), 0.25 g of yeast powder (Oxiod, Basingstoke, UK), 0.2 g of sodium pyruvate (Amresco, Boise, ID, USA), 20 mL of horse serum (Solarbio, Beijing, China), 8000 IU of penicillin G (BioFroxx, Einhausen, Germany), and 250 µL of 0.4% phenol red solution (Amresco, Boise, ID, USA). Mycoplasma solid medium was prepared by adding 1.5% agar powder (BioFroxx, Einhausen, Germany) to the mycoplasma complete medium formula (without phenol red). One hundred microliters of the joint effusion were aspirated, added to 900 µL of sterile PBS (Gibco, Jenks, OK, USA), and mixed by pipetting. This operation was repeated thrice to dilute to 10^−4^ of the stock solution. Fifty microliters of joint effusion at different dilutions was, respectively, inoculated into a mycoplasma complete medium and a mycoplasma solid medium. Then we placed them in a 37 °C, 5% CO_2_ incubator for culturing and observed every day for recording.

When turbidity or color changes occurred in the mycoplasma complete medium, we used a 0.45 μm sterile filter membrane (Merck, Darmstadt, Germany) to filter the culture fluid. The filtered broth was then inoculated into fresh mycoplasma complete medium at a volume ratio of 1:10 for passaging. After three passages following the above protocol, the purified broth was obtained. Mycoplasma solid medium requires observation every 1–2 d using an ordinary optical microscope (Olympus, Tokyo, Japan) after inoculation. If a similar colony is observed, it must be clonally purified promptly. Subsequently, purified colonies on the plates were stained with Giemsa.

Finally, the purified colonies were identified by PCR. At the same time, the Mmc PG3 strain was used as a positive control. Relevant identification primers for the bacterial 16S rRNA gene and the *Mmc 3740* gene were designed (Table 1) [16,17]. The PCR mixture included 1 µL of DNA template, 10 pmol of upstream primer, 10 pmol of downstream primer, 7 µL of ddH_2_O, and 10 µL of 2 × Taq PCR mix. The PCR procedure (Bio-Rad, Hercules, CA, USA) included pre-denaturation at 94 °C for 5 min, denaturation at 94 °C for 30 s, annealing temperature as shown in Table 1, extension at 72 °C for 90 s, cycle for 30 times, and final extension at 72 °C for 10 min.

### 2.2. Culture and DNA Extraction of Mmc HN-A

The Mmc HN-A strain was inoculated into 200 mL of PPLO broth (containing 0.6 g of glucose, 0.5 g of yeast powder, 40 mL of horse serum, 160,000 IU of penicillin, 0.4 g of sodium pyruvate, and 5000 μL of 0.4% phenol red) for culture at a volume ratio of 1:100.

The Mmc HN-A genome was extracted using a HiPure Bacterial DNA Kit (Magen, Guangzhou, China). The concentration of extracted genomic DNA was measured using a Qubit (Thermo Fisher Scientific, Waltham, MA, USA). Finally, the extracted genomic DNA purity was checked using a Nanodrop spectrophotometer (Thermo Fisher Scientific, Waltham, MA, USA). Only when both indicators were qualified could the genomic DNA be interrupted for library construction.

### 2.3. PacBio Sequencing

An SMRTbell library was generated from sheared genomic DNA using a template library preparation workflow with protocols and reagents according to the manufacturer’s instructions (PacBio, Menlo Park, CA, USA). After the library was constructed, a Qubit^®^ 2.0 Flurometer (Life Technologies, Carlsbad, CA, USA) was used for quality checking, and a Bioanalyzer 2100 (Agilent, Santa Clara, CA, USA) was used to evaluate the average fragment size. PacBio sequencing was performed using the Pacific Biosciences Sequel sequencer (PacBio, Menlo Park, CA, USA).

### 2.4. Illumina Sequencing

Following DNA quality testing, the extracted DNA was used to construct libraries for Illumina Sequence by Synthesis genome sequencing. DNA libraries were prepared using the NEBNext^®^ Ultra™ II DNA Library Prep Kit for Illumina^®^ (NEB, Ipswich, MA, USA) and genomic sequencing was performed using an Illumina Novaseq 6000 sequencer. Briefly, the genomic DNA was fragmented, followed by end-repair, A-tailing, adaptor ligation, and library amplification according to the manufacturer’s instructions. DNA fragments with a length of 300–400 bp were purified with AMPure XP magnetic beads (Beckman Coulter, Brea, CA, USA). The DNA library was evaluated using a 2100 Bioanalyzer (Agilent, Santa Clara, CA, USA) and real-time PCR for quality control analysis. Finally, the library was subjected to paired-end sequencing (PE150).

### 2.5. Genome Assembly

In this study, the Mmc HN-A genome was sequenced by combining PacBio and Illumina sequencing. Relying on the characteristics of PacBio sequencing long read length guarantees more complete genome assembly. Illumina sequencing data were used for correction to ensure that assembly results were more precise and reliable.

Raw PacBio and Illumina sequencing data were filtered to obtain clean data. The filtered reads were then assembled de novo to generate one contig without gaps using Falcon (version 0.3.0, Victoria, BC, Canada) [18]. Illumina sequencing data were screened using FASTP (version 0.20.0, London, UK) [19]. Clean Illumina sequencing data were aligned to the assembled genome sequence using Pilon (version 1.23, Bracknell, UK) [20]. Finally, the genomic results were corrected using default software parameters to obtain the final genomic sequence. The circle map of the Mmc HN-A genome was drawn using Circos (version 0.69–9, San Francisco, CA, USA) [21].

### 2.6. Genome Annotation and Bioinformatic Analysis

A bioinformatics analysis of the whole-genome sequence was performed, including the prediction of coding sequences, repetitive sequences, non-coding RNA, genomic islands (GIs), prophages, transposons, and clustered regularly interspaced short palindromic repeat sequences (CRISPR). The NCBI prokaryotic genome annotation pipeline [22] was used to predict coding sequences, whereas the interspersed repetitive sequences were predicted using RepeatMasker (version open-4.0.5, Institute for System Biology (ISB, Seattle, WA, USA) [23]. Tandem repeats were analyzed using Tandem Repeats Finder TRF (version 4.09, Benson Genomics Lab, New York, NY, USA) [24]. Transfer RNA (tRNA) genes and ribosomal RNA (rRNA) genes were predicted using tRNAscan-SE (version 1.3.1, University of California, Santa Cruz, CA, USA) [25] and RNAmmer (version 1.2, DTU Health Tech, Lyngby, Denmark) [26], respectively. Small RNAs (sRNAs) were predicted using cmscan (version 1.1.2, Howard Hughes Medical Institute, Chevy Chase, MD, USA) [27] blast analysis against the Rfam database. IslandPath-DIMOB (version 1.0.0, Simon Fraser University, Burnaby, BC, Canada) [28] was used to identify gene islands, whereas PHAST (version 2.0, DNV, Bærum, Norway) [29] was used to predict the prophage locations. CRISPR and transposons were identified using CRISPRfinder (version 4.2.17, Institut de Génétique et Microbiologie, Orsay, France) [30] and TransposonPSI (version 20100822, Broad Institute, Cambridge, MA, USA) [31].

### 2.7. Gene Function Analysis

We used several complementary methods to annotate the assembled sequences. Four databases were used to predict gene functions, including the Gene Ontology database (GO) [32], UniProt/Swiss-Prot, Kyoto Encyclopedia of Genes and Genomes (KEGG) database [33], Clusters of Orthologous Groups (COG) database [34], and Non-redundant Protein (NR) Database [35]. A whole-genome blast search (E-value < 1 × 10^−5^, minimum alignment length percentage > 40%) was performed against each of the four databases.

Secreted proteins were predicted using SignalP4.0 [36], while EffectiveT3 (version 1.0.1, University of Vienna, Wien, Austria) [37] was used to predict type three secretion systems (T3SS). The Pathogen–Host Interactions (PHI) database [38] and Virulence Factors of Pathogenic Bacteria database (VFDB) [39] were used to identify virulence factors. The target sequences were aligned with the Pfam database (version 32.0, EMBL-EBI, Hinxton, UK) using the default parameters of Pfam_Scan (version 1.6, EMBL-EBI, Hinxton, UK) [40] for protein family queries and protein domain annotations. The protein sequence containing the signal peptide was analyzed using TMHMM (version 2.0, DTU Health Tech, Lyngby, Denmark) [41] to identify the transmembrane protein. The gene sequences were aligned and annotated with the carbohydrate-active enZYmes database (CAZy) using blastp. Two-component systems (TCSs) in bacterial genomes were predicted based on the characteristics of TCSs. The predicted gene sequences were aligned to the Comprehensive Antibiotic Resistance Database (CARD) for annotation using a resistance gene identifier (RGI). Secondary metabolic gene clusters were predicted using antiSMASH (version 4.1.0, DTU Health Tech, Lyngby, Denmark) [42].

Comparative genomic analysis was performed using MUMmer [43] (Version 3.1, University of Hamburg, Hamburg, Germany), and samples were detected for structural variants using SyRI [44] (Version 1.4). We selected 4 genomes of species with close genomic proximity to the Mmc HN-A strain to carry out gene family analysis using Diamond [45] (Version 2.0.7, Crystal Impact GbR, Bonn, Germany) and OrthoMCL [46] (Version 1.4). The screening was performed by using the criterion of 40% amino acid similarity over 80% length in the shortest protein sequence.

### 2.8. Phylogenetic Tree Construction

Genes in gene families that were single copies in both the Mmc HN-A and reference genomes were selected. The evolutionary relationships among species were investigated using IQ-TREE [47] (version 1.6.3, Australian National University, Canberra, Australia) and the maximum likelihood method was used for constructing phylogenetic trees. Based on the collinear alignment completion, pyani [48] (Version 0.2.7, Massachusetts Institute of Technology, Cambridge, MA, USA) was used to calculate the average nucleotide identity (ANI) for genomic aligned regions between the Mmc HN-A genome and reference genome to determine their relatedness.

## 3. Results and Discussion

### 3.1. Results of Isolation and Identification of Mmc HN-A

**Figure 1:**
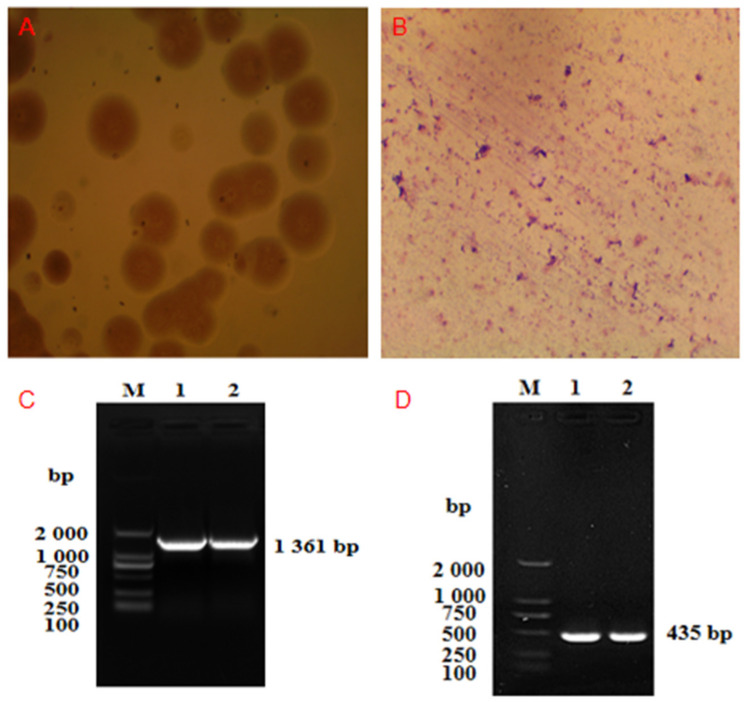
Isolation and identification of Mmc HN-A. (**A**), Colonies of isolated strains were observed under a microscope at low magnification (40×); (**B**), Giemsa staining results of isolated strains (1000×); (**C**), PCR identification of the 16S rRNA gene: (M) D2000 Marker, (Line 1) the isolated strain, (Line 2) the Mmc PG3 strain; (**D**), PCR identification of the *Mmc 3740* gene: (M) D2000 Marker, (Line 1) the isolated strain, (Line 2) the PG3 strain.

We successfully isolated and purified this strain. The purified colonies were needle tip-sized, white in color, and water droplet-type. When magnified by 40 times under an ordinary optical microscope, it can be observed that the shapes and sizes of colonies were different. Most colonies were round or oval in shape and had a central umbilicus (Figure 1A). After the Giemsa staining of the isolated strain, purple particulate matter was observed under an oil microscope. It has various shapes, such as globular and arc-shaped (Figure 1B).

For the isolated strain, the 1361 bp product was successfully amplified by PCR with the 16S rRNA primers in Table 1 (Figure 1C). The PCR products were recycled after gel electrophoresis and sent to Sangon Biotech Co., Ltd. (Shanghai, China), for sequencing. The sequencing results were input into NCBI for blast. It was found that the sequence homology of the 16S rRNA gene between the isolated strain and the Mmc PG3 strain was 99.93%. Therefore, the strain can be preliminarily determined as Mmc. We selected a specific Mmc gene for PCR identification (Figure 1D). Eventually, we confirmed that the isolated strain was Mmc and named it the “Mmc HN-A strain”.

Hainan Island is located northwest of the South China Sea and faces the Leizhou Peninsula across the Qiongzhou Strait in the north. It is China’s second largest island and the main subject of China’s southernmost provinces. We isolated and identified Mmc for the first time on Hainan Island in this study, which filled the information on local epidemic Mmc strains and had a positive impact on local ruminant farming.

### 3.2. General Features of Mmc HN-A

**Figure 2:**
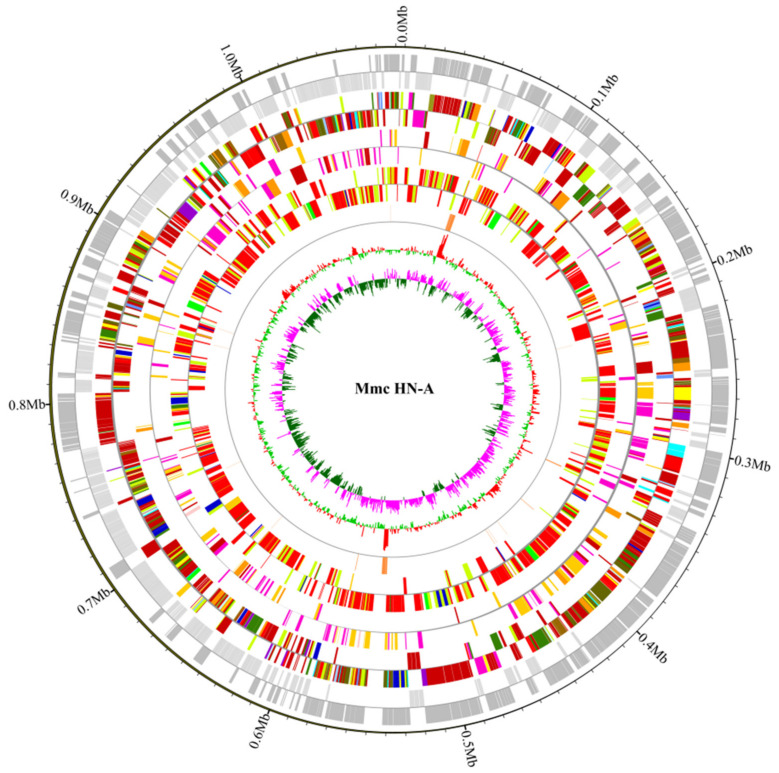
Whole genome map of Mmc HN-A. The outermost circle represents the positional coordinates of genome sequences. From the outer circle to the inner circle are coding genes, COG database annotation results, KEGG database annotation results, GO database annotation results, ncRNA, GC content, and GC skew (GC offsets were used to mark start and end points in circular chromosomes; both the window and step size were 1000).

We obtained 0.90 Gb (~833×) and 1.05 Gb (~972×) data from the PacBio (Menlo Park, CA, USA) and Illumina (San Diego, CA, USA) sequencing platforms, respectively. These data were used for de novo assembly to generate a circular Mmc-HN-A genome map. The genome-wide map of Mmc HN-A (Figure 2) was mapped using Circos software based on information such as basic sequence information, GC content, coding genes, non-coding RNAs, and the COG functional classification of genes so that we were able to attain a more comprehensive and intuitive view of the Mmc HN-A genome characteristics. The annotated whole-genome sequence of Mmc HN-A has been deposited in the GenBank database under the accession number CP093215.

The Mmc HN-A genome contains a circular chromosome of 1,084,691 bp with a GC content of 23.76%. It contains two gene islands, 848 coding genes, and three DNA transposons. The Mmc HN-A genome also contains three transposons, chain00001, chain00002, and chain00003, whose types are ltr_Roo, TY1_Copia, and helitron ORF (Table S1). The chain00003 is transposed by rolling circle replication, which can capture and carry gene fragments, thereby causing changes in the gene copy number. Additionally, the five potential CRISPRs were predicted in Mmc for the first time (Table S2). CRISPR is a natural bacterial immune system that can resist foreign plasmids and phage sequences and silence invading functional elements. The general characteristics of the Mmc HN-A genome were shown in Table 2.

### 3.3. Functional Annotation of Mmc HN-A

The Mmc HN-A genome was compared and analyzed using different functional databases, and the alignment results with the highest scores were selected for annotation. The functional annotation results for the Mmc HN-A genome were presented in Supplementary Figure S3. A total of 418 genes were simultaneously annotated in all four databases (Figure S3 and Table S3), and the specific annotation information was presented in Supplementary Figure S1. Additionally, we predicted that the Mmc HN-A genome did not contain secondary metabolic gene clusters or two-component systems.

Secreted proteins include many common small molecule metabolites and pathogenic factors. Therefore, they were predicted to be important for studying pathogenic bacteria. The Mmc HN-A genome encodes 848 proteins, of which 70 were predicted to have signal peptide structures (SignalP) (Table S4). Among the 70 SignalP proteins, 13 were transmembrane structures, and the remaining 57 were secreted proteins (Tables S5 and S6).

#### 3.3.1. Virulence Factors

Virulence factors are an important part of Mmc and play an important role in host infection and cell colonization. However, as the smallest self-replicating class of microorganisms, Mmc has received limited research on its virulence factors. Therefore, a virulence gene analysis of the Mmc HN-A strain was performed using PHI, VFDB, and CARD databases.

**Figure 3:**
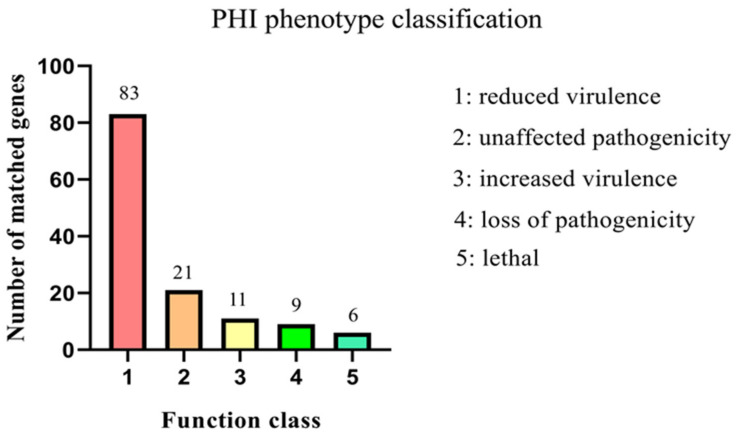
PHI database annotation results of the Mmc HN-A genome. Note: The abscissa indicates the type of phenotypic mutation. The ordinate indicates the number of genes.

The PHI database contains many experimentally verified sequences of pathogenic and effector genes, which are often used for pathogenic gene analysis. We performed PHI annotation on the Mmc HN-A genome, and the results were as follows: In the Mmc HN-A genome, 130 genes were annotated using the PHI database. Among them, 11 genes belonged to the type of increased virulence group, which are key pathogenic genes. However, the specific functions of virulence genes require further experimental verification. (Figure 3 and Table S7).

The Mmc HN-A amino acid sequences were aligned in the VFDB database using the Diamond software, and 16 genes were annotated (Table S8). Most genes were annotated as capsule genes. Only one gene was annotated as ef-TU, PDH-B, hemolysin, streptococcal enolase, and cereulide. MNF30_03455 and MNF30_00555 were annotated as surface lipoproteins.

We identified virulence factors, such as GlF (MNF30_00985), PdhB (MNF30_03905), HlyA (MNF30_04500), TuF (MNF30_04280), and two variable surface proteins vmm (Vmm) (MNF30_00555 and MNF30_03455), which are highly similar to Vmm (MSC_0979) of the MmmSC PG1 strain. Of particular concern were *glf* (*MNF30_00985*) and *hlyA* (*MNF30_04500*). In previous studies, *glf* was shown to be a virulence gene of Mmc. In the absence of *glf*, the strain showed a reduced growth rate, decreased cell membrane integrity, and increased adhesion to small ruminant cells. The effect of goat serum antibody on the deletion strain was weakened [49]. Hemolysin A can damage and lyse red blood cells [50], promote small vessel smooth muscle contraction, and cause capillary blockage or ischemic necrosis. Li et al. [51] identified *hlyA* (*MCCG_0074*) in the Mccp 87001 genome. In this study, we identified *hlyA* (*MNF30_04500*) in the Mmc HN-A genome for the first time, and its sequence was highly homologous to that of the Mcc ATCC 27343 strain.

Simultaneously, thirty-eight genes in Mmc HN-A were annotated in the CARD database (Table S9). We found that the Mmc HN-A strain may produce resistance to macrolides, fluoroquinolones, tetracyclines, streptomycin, and other antibiotics through the resistance mechanism of antibiotic efflux and antibiotic target alteration. This study provides a reference for clinical diagnosis and medication.

**Figure 4:**
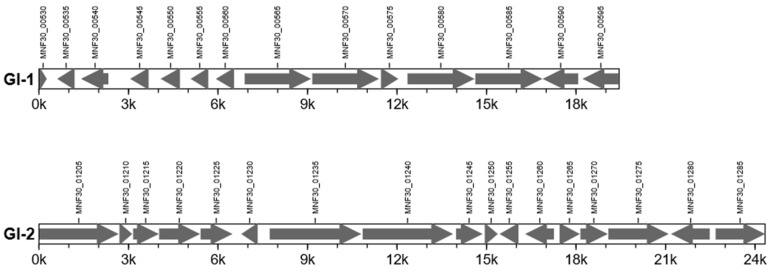
Gene distribution map in the Mmc HN-A gene island. Note: The abscissa is the length scale.

Additionally, secretion systems and gene islands also affect bacterial virulence. Therefore, we used Effective T3 software to predict the bacterial type IV secretion system protein VirD4. The protein is encoded by *MNF30_03290* and has a KO ID of ko03070, which plays a role in membrane transport and environmental information processing of Mmc, and is closely related to bacterial pathogenicity. For the first time, we identified two gene islands, GI_1 and GI_2, in Mmc HN-A (Figure 4 and Table S10). They contained a total of 31 genes, of which GI_1 contained 14 genes and GI_2 contained 17 genes. GI_1 belongs to the pathogenic gene island, which is closely related to Mmc HN-A virulence. GI_1 encodes five lipoproteins: MNF30_00535, MNF30_00545, MNF30_00550, MNF30_00555, and MNF30_00560. The virulence factor encoded by *MNF30_00555* is a surface lipoprotein. At the same time, *MNF30_00530* in GI_1 is a unique gene of the Mmc HN-A strain, and its encoded hypothetical protein requires further study. The IS3 family transposase encoded by *MNF30_00540* in GI_1 and the transposase encoded by *MNF30_01250* in GI_2 provide the possibility for the Mmc HN-A genome evolution.

#### 3.3.2. Metabolism

There were 22 genes involved in the glycolysis metabolic pathway in the Mmc HN-A genome. Simultaneously, there is an oligopeptide transport system comprising ten genes, a nucleoside transport system comprising four genes, and a phosphonate transport system comprising six genes in the genome.

The phosphotransferase system (PTS) is widely found in archaea and prokaryotes and usually comprises EI, HPR, and EⅡ [52]. In this study, we predicted 19 genes to be involved in the Mmc HN-A strain PTS system (Table S11). Among them, *MNF30_03870* and *MNF30_01490* encoded the cytosolic soluble type proteins, EI and HPR, respectively. The EⅡA^Glc^ and EⅡCB^Glc^ proteins are, respectively, encoded by *MNF30_03865* and *MNF30_00775*, which play important roles in glucose phosphorylation transport. Through the Mmc HN-A strain PTS system, fructose can be metabolized to generate fructose 1,6-bisphosphate, which may replace glucose as the carbon source for energy supply. Additionally, maltose and trehalose can also be metabolized to generate maltose 6′-phosphate and trehaloss 6-phosphate, respectively. This provides a reference for optimizing and improving the culture medium for the Mmc HN-A strain.

Additionally, *MNF30_04105* encodes oligoendopeptidase F and *MNF30_00320* encodes an ATP-dependent metallopeptidase. S41 peptidase is associated with the proteolytic phenotype of the strain and is encoded by *MNF30_03590* and *MNF30_03835*.

### 3.4. Results of Comparative Genomic Analysis

#### 3.4.1. Collinearity Analysis Results of The Five Strains

Mmc GM12, Mmc Ker.TCR. LT, MmmLC 95010, and MmmSC PG1 were selected as the target genomes and Mmc HN-A was used as the reference genome (Table 3). Using the collinearity analysis method, a parallel collinearity diagram was drawn to compare the evolutionary distance difference between the two species. Then, the genetic relationship between the sample species and the reference species was analyzed.

**Figure 5:**
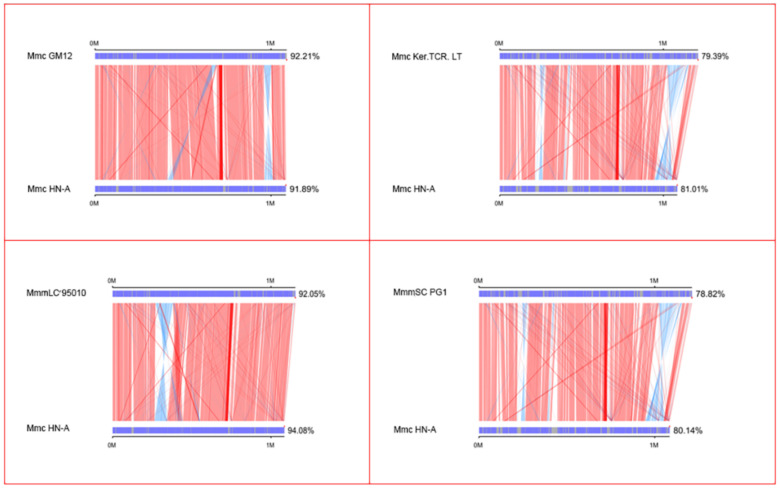
Parallel collinearity result plot. The upper axis is the target species genome; the lower axis is the reference genome; the red line represents the forward matching of the corresponding region; the blue line represents the reverse matching of the corresponding region.

The results showed that the alignment sequence region of Mmc HN-A with Mmc GM12 and MmmLC 95010 accounted for more than 90% of the entire genome, and the number of alignment blocks was not more than 20 (Figure 5). This indicated that the evolutionary distance between them was relatively close. The aligned sequence region of Mmc HN-A with Mmc Ker.TCR. LT and MmmSC PG1 was less than 82% of the entire genome, and there was a similar large reverse matching region. Thus, they are far from evolutionary distance from Mmc HN-A.

#### 3.4.2. Statistical Results of SNP, InDel, and SV

We counted the type and number of genomic structural variations in the Mmc HN-A. Compared with the genomes of Mmc Ker.TCR. LT and MmmSC PG1, the number of insertions, deletions, and SV in Mmc HN-A was higher than those of Mmc GM12 and MmmLC 95010. This was particularly evident for differences in the number of SVs (Table 4).

In the variant-type display diagram (Figure S2), we determined that the Mmc HN-A genome structure was closer to that of Mmc GM12 and MmmLC 95010. At the same time, a similar large reverse matching region of Mmc Ker.TCR. LT and MmmSC PG1 in 3.4.1 was identified as an inversion region. The overall trends of all variant types of Mmc Ker.TCR. LT and MmmSC PG1 were consistent, which is different from those of Mmc GM12 and MmmLC 95010.

### 3.5. Gene Family Analysis and Pan-Genome Analysis Results of Five Strains

Genes within a family have similar structures and functions. Through gene family analysis, we were able to determine the structure and function of unknown genes. We identified 33 unique Mmc HN-A gene families, including 36 specific genes (Table S12). The specific Mmc HN-A genes encode nine hypothetical proteins, one ABC transporter, and three lipoproteins (*MNF30_00070*, *MNF30_00080*, and *MNF30_03445*) (Table 5 and Table 6). This study provides a basis for studying the environmental adaptation and virulence of Mmc HN-A.

Additionally, these specific genes encode proteins related to type I RM system. The *MNF30_00015* and *MNF30_00030* genes contained in ORTHOMCL912 encode HsdS protein. The *MNF30_00035* and *MNF30_02040* genes contained in ORTHOMCL913 encode HsdM protein. *MNF30_00025* and *MNF30_02035* encode HsdR (K01153). The type I RM system is ubiquitous in bacteria and it is considered as the most primitive immune system of bacteria [53]. Studies have shown that the type I RM system can regulate the expression of bacterial virulence genes and affect the virulence of bacteria [54,55]. Therefore, we speculated that the above genes in the type I RM system are essential for specific Mmc HN-A virulence.

**Figure 6:**
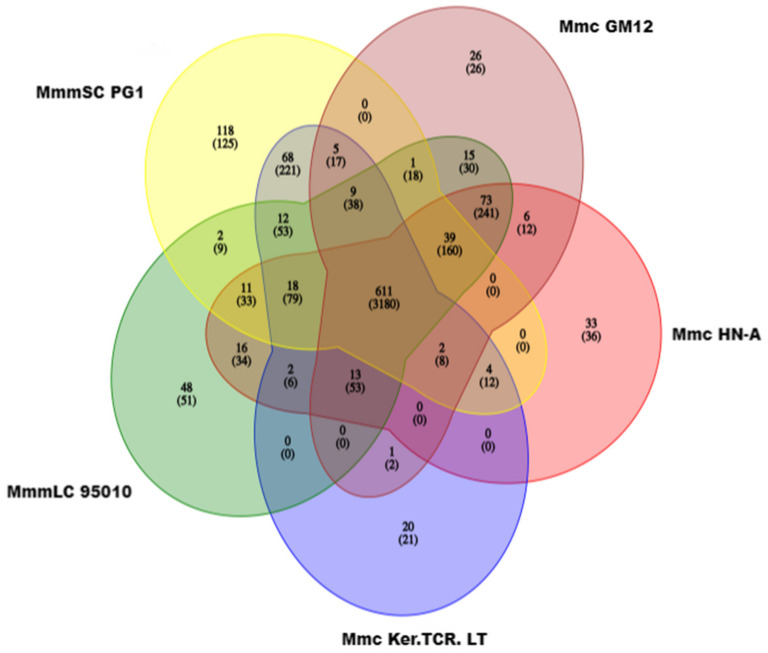
Homologous gene families of five *Mycoplasma mycoides* strains. Each ellipse represents a genome. The number above each region indicates the number of gene families in each species. The number in the lower brackets indicates the total number of genes in the gene families.

The pan-genome of the five *Mycoplasma mycoides* contained 1153 genes, of which the core genome contained 611 genes. These 611 genes encoded genes that expressed the core biological functions and major phenotypic features of *Mycoplasma mycoides*. For example, the *MNF30_02070* and *MNF30_02080* genes in ORTHOMCL3 are shared by five strains and are related to the pathogenic mechanism of *Mycoplasma mycoides*. It can provide a reference for virulence research and vaccine design for *Mycoplasma mycoides* (Table 6 and Figure 6).

### 3.6. Phylogenetic Tree Analysis and ANI Analysis

**Figure 7:**
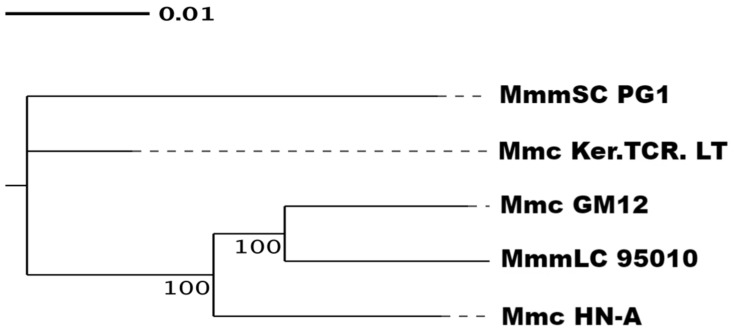
Species evolution tree based on characteristic genes. The phylogenetic tree shows the evolutionary relationships among different species and their topology. The end of the tree diagram is for each species. The intersection point is the common ancestor between species, and the branch length indicates the relative genetic distance between the species. The number on the branch indicates its reliability.

The genomes of these five mycoplasma strains were highly similar, and the genetic relationships between the strains could not be accurately identified by collinearity analysis of the genomes. Therefore, we used phylogenetic tree and ANI analysis to further determine the genetic evolutionary distances between the strains. Single copies of homologous genes were selected from homologous gene family clustering for multiple sequence alignment and quality control using MUSCLE [56] software (EMBL-EBI, Hinxton, UK) and Gblocks [57] software (IDT, Coralville, IA, USA), respectively. A phylogenetic tree was constructed using the single-copy gene method (Figure 7). Mmc HN-A had the closest genetic distance to that of MmmLC 95010, followed by Mmc GM12.

**Figure 8:**
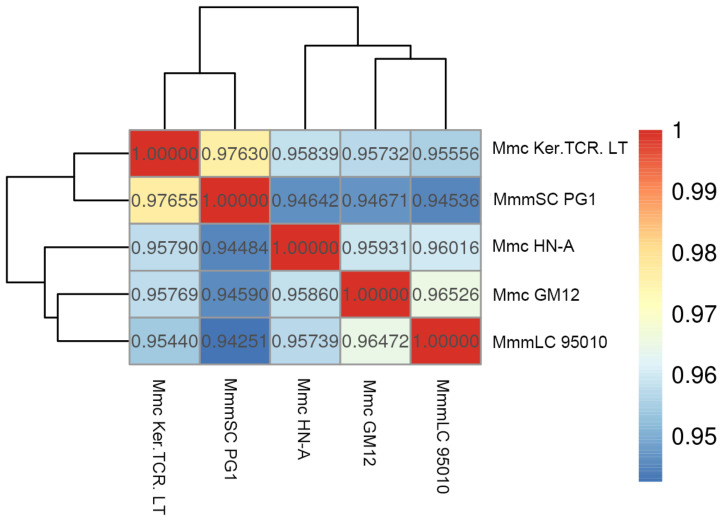
ANI Analysis Heatmap.

The ANI value refers to the average base similarity between homologous fragments of two microbial genomes and usually discriminates against different species with 95.00% as a classification threshold, which has high discrimination among close relatives. Based on the collinear alignment completion, the ANI values were calculated to construct a heat map (Figure 8). The results showed that, compared with Mmc HN-A, the largest ANI value was MmmLC 95010. This shows that Mmc HN-A has the closest genetic relationship to MmmLC 95010. Interestingly, the ANI values between Mmc Ker.TCR. LT and MmmSC PG1 were the highest, indicating that they were closely related. This suggests that ANI analysis has certain limitations. In conclusion, we identified MmmLC 95010 and Mmc GM12 as having the closest genetic relationships to Mmc HN-A. Mmc Ker.TCR. LT had the closest genetic distance to the MmmSC PG1.

## 4. Conclusions

Mmc, MmmSC, and MmmLC were preliminary considered as three different subspecies of *Mycoplasma mycoides*. Subsequently, MmmLC was reclassified as an Mmc serotype in 2009. Therefore, *Mycoplasma mycoides* contains only two subspecies, Mmc and MmmSC, nowadays. Their hosts are sheep and cattle. In this study, the Mmc HN-A strain was isolated and identified for the first time in Hainan Island, China. The genomic analysis of Mmc HN-A revealed the molecular characteristics of strains, such as virulence factors, gene islands, and antibiotic resistance, which provides a reference for later drug development and vaccine design. Meanwhile, the core genome of *Mycoplasma mycoides* was identified by comparative genomic analysis between Mmc HN-A and the other four strains, which provides a new perspective for understanding the core biological functions and the main characterization of *Mycoplasma mycoides*. The shared and specific genes contained in Mmc, MmmSC, and MmmLC were analyzed, which provides a reference for studying the differentiation and characteristics of different *Mycoplasma mycoides* subspecies. Finally, we showed the evolutionary relationships between different subspecies within *Mycoplasma mycoides* by constructing a phylogenetic tree of single-copy homologous genes.

## Figures and Tables

**Table 1:** Related primer information of bacteria.

Detection Object	Primer Name	Primer Sequence (5′-3′)	Product Size (bp)	Annealing Temperature (°C)
Bacterial 16S rRNA	16S rRNA	F: AGAGTTTGATCCTGGCTCAG	1465	56
		R: GGTTACCTTGTTACGACTT		
Mmc	Mmc 3740	F: GGATCCGTGATAAAGTTATT GACTTTATTAG	435	50
		R:AAGCTTTTATTGTACTGTATTGTGTTCTTC		

**Table 2:** General characteristics of Mmc HN-A.

Item	Number	Item	Number
Genome size (bp)	1,084,691	Number of genes	848
Total length of gene (bp)	966,987	Average length of gene (bp)	1,140.31
Genome GC content (%)	23.76	Total length of gene/genome (%)	89.15
GC content of gene region (%)	23.89	Total length of intergenic region/genome (%)	10.85
The length of gene (bp)	75–9840	Tandem repeat length/genome (%)	0.62
Number of tandem repeats	146	Number of gene islands	2
Total bases in tandem repeats	6747	Total length of gene island (bp)	43,779
Number of interspersed repeats	8	Number of short-interspersed elements	5
Number of rRNA genes	6	Number of DNA elements	3
Number of tRNA genes	30	Total potential CRISPRs	5
Number of sRNA genes	2	Number of Prophages	0

**Table 3:** Basic information of the five *Mycoplasma mycoides* strains.

	Mmc HN-A	Mmc GM12	Mmc Ker.TCR. LT	MmmLC 95010	MmmSC PG1
Accession number	CP093215	CP001621.1	CP068548.1	FQ377874.1	BX293980.2
Place of isolation	China	USA	India	France	Sweden
Collection date	2021	2009	2019	1995	2003
Size (bp)	1,084,691	1,089,202	1,211,756	1,153,998	1,211,703
G + C (%)	23.76	23.9	24.0	23.81	24.0

**Table 4:** Statistical results of SNP, InDel, and SV between Mmc HN-A and target strains.

	SNPs	Insertion	Deletion	SV
Mmc GM12	25,297	566	540	51
Mmc Ker.TCR. LT	19,624	605	676	98
MmmLC 95010	26,003	520	527	68
MmmSC PG1	27,246	574	657	101

**Table 5:** Gene family analysis of five *Mycoplasma mycoides* strains.

Species	Total Genes	Gene in Families	Unclustered Genes	Families	Unique Families
Mmc Ker.TCR. LT	847	828	19	746	20
MmmLC 95010	922	877	45	825	48
MmmSC PG1	1016	905	111	789	118
Mmc GM12	832	806	26	775	26
Mmc HN-A	848	818	30	798	33

**Table 6:** Specific genes of five *Mycoplasma mycoides* strains.

Species	Total Genes	Shared Gene Number	Specific Gene Number
Mmc Ker.TCR. LT	847	826	21
MmmLC 95010	922	871	51
MmmSC PG1	1016	891	125
Mmc GM12	832	806	26
Mmc HN-A	848	812	36

## Data Availability

The annotated whole-genome sequence of Mmc HN-A has been deposited in the GenBank database under the accession number CP093215. All the analyzed datasets in the current study are available from the corresponding author on reasonable request.

## Supplementary Materials

The following supporting information can be downloaded at: https://www.mdpi.com/article/10.3390/microorganisms10101908/s1, Figure S1: Annotation information of Mmc HN-A in four major databases. (A) Species statistics were annotated using the NR database, where the abscissa represents the species ID. The ordinate axis represents the number of genes annotated. (B) GO functional classification map of the Mmc HN-A genome. The abscissa represents annotated GO terms. The ordinate represents the number of genes annotated for each GO term. (C) Classification diagram of the KEGG metabolic pathway of Mmc HN-A. The horizontal axis indicates the number of genes. The ordinate axis represents the functional classification of the KEGG database. (D) COG function classification diagram of Mmc HN-A. The horizontal axis represents the COG function type. The ordinate indicates the number of annotated genes; Figure S2: Variation type display diagram. (A) Variation type display diagram of Mmc GM12 vs Mmc HN-A. (B) Variation type display diagram of MmmSC PG1 vs Mmc HN-A. (C) Variation type display diagram of Mmc Ker.TCR. LT vs Mmc HN-A. (D) Variation type display diagram of MmmLC 95010 vs Mmc HN-A; Figure S3: Genes annotation statistics of Mmc HN-A. (A) Venn diagram of the four major databases annotation. (B) Genes annotation statistics of Mmc HN-A.; Table S1: Transposons in Mmc HN-A; Table S2: The potential CRISPRs in Mmc HN-A; Table S3: Intersection of four database annotation results for Mmc HN-A; Table S4: SignalP proteins in Mmc HN-A; Table S5: Proteins with transmembrane structure in Mmc HN-A; Table S6: Secreted proteins in Mmc HN-A; Table S7: PHI database annotation results of Mmc HN-A; Table S8: VFDB database annotation results for Mmc HN-A; Table S9: CARD database annotation results of Mmc HN-A; Table S10: Gene islands of Mmc HN-A; Table S11: Phosphotransferase system of Mmc HN-A; Table S12: Specific genes of Mmc HN-A.
