# Supplementary material for: First Report and Comparative Genomic Analysis of a Mycoplasma mycoides Subspecies capri HN-A in Hainan Island
Source: Microorganisms. 2022 Sep 26;10(10):1908. doi: 10.3390/microorganisms10101908 (PMC9607973; doi:10.3390/microorganisms10101908)
Supplement: Supplementary file 1 [file microorganisms-10-01908-s001.zip › Figure S1. Annotation information of Mmc HN-A in four major databases.pdf]

Figure S1

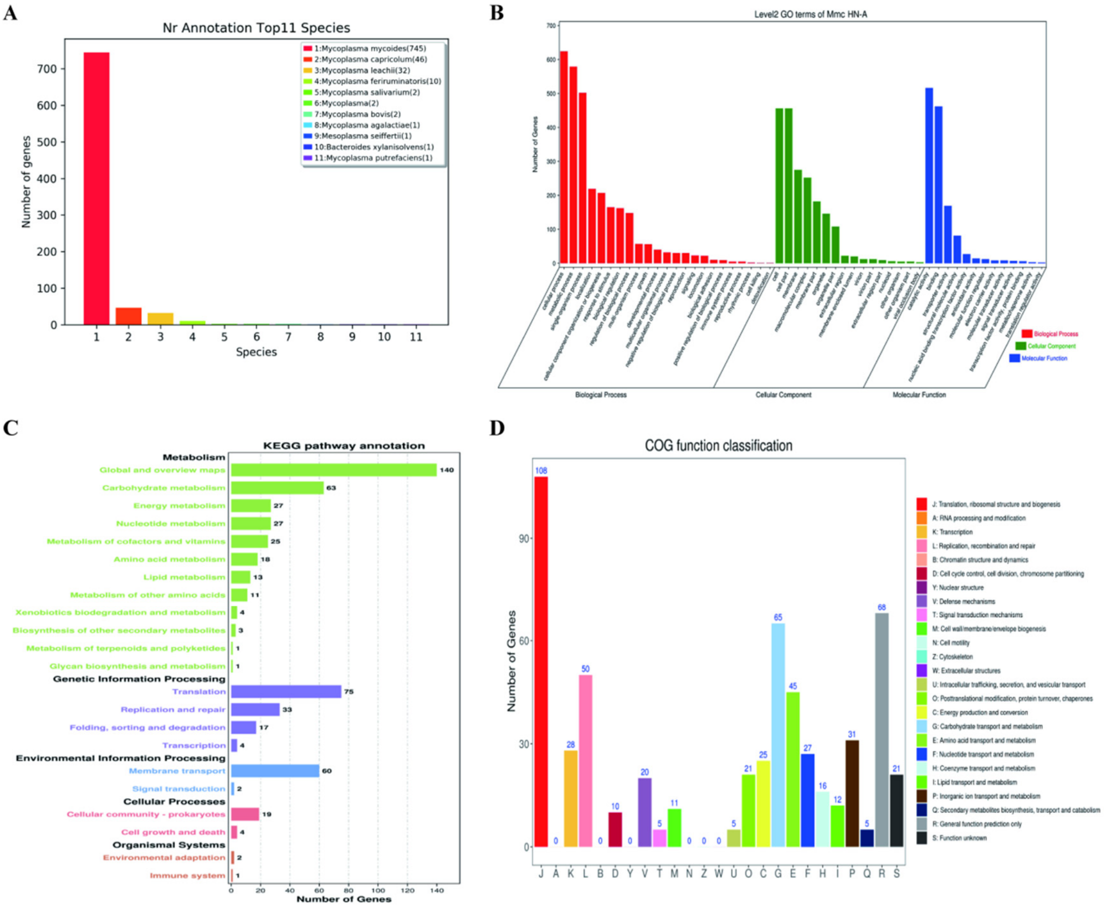

Figure S1. Annotation information of Mmc HN-A in four major databases.

(A) Species statistics were annotated using the NR database, where the abscissa represents the species ID. The ordinate axis represents the number of genes annotated.
(B) GO functional classification map of the Mmc HN-A genome. The abscissa represents annotated GO terms. The ordinate represents the number of genes annotated for each GO term.
(C) Classification diagram of the KEGG metabolic pathway of Mmc HN-A. The horizontal axis indicates the number of genes. The ordinate axis represents the functional classification of the KEGG database.
(D) COG function classification diagram of Mmc HN-A. The horizontal axis represents the COG function type. The ordinate indicates the number of annotated genes.
