# Supplementary material for: First Report and Comparative Genomic Analysis of a Mycoplasma mycoides Subspecies capri HN-A in Hainan Island
Source: Microorganisms. 2022 Sep 26;10(10):1908. doi: 10.3390/microorganisms10101908 (PMC9607973; doi:10.3390/microorganisms10101908)
Supplement: Supplementary file 1 [file microorganisms-10-01908-s001.zip › Figure S2. Variation type display diagram.pdf]

Figure S2. Variation type display diagram

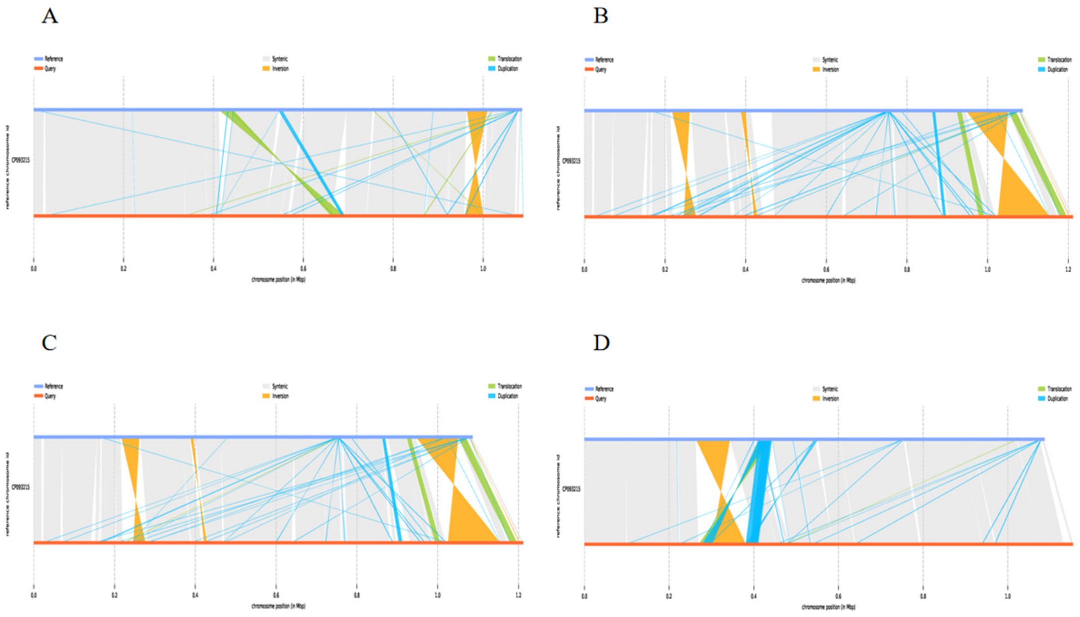

Figure S2. Variation type display diagram
(A) Variation type display diagram of Mmc GM12 vs Mmc HN-A
(B) Variation type display diagram of MmmSC PG1 vs Mmc HN-A
(C) Variation type display diagram of Mmc Ker.TCR. LT vs Mmc HN-A
(D) Variation type display diagram of MmmLC 95010 vs Mmc HN-A
