# Supplementary material for: First Report and Comparative Genomic Analysis of a Mycoplasma mycoides Subspecies capri HN-A in Hainan Island
Source: Microorganisms. 2022 Sep 26;10(10):1908. doi: 10.3390/microorganisms10101908 (PMC9607973; doi:10.3390/microorganisms10101908)
Supplement: Supplementary file 1 [file microorganisms-10-01908-s001.zip › Figure S3. Genes annotation statistics of Mmc HN-A.pdf]

Figure S3 Genes annotation statistics of Mmc HN-A

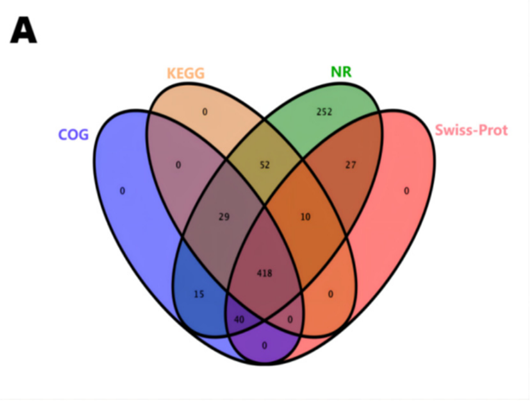

B

Database	Number of annotated genes
GO	731
KEGG	509
COG	502
NR	843
Secretory_Protein	57
Swiss-Prot	495
PHI	130
VFDB	15
CARD	38
T3SS	1
CAZy	92

Figure S3 Genes annotation statistics of Mmc HN-A

(A) Venn diagram of the four major databases annotation

(B) Genes annotation statistics of Mmc HN-A.
